# The persistent problem of integrated care in English NHS hospitals: Is the Mayo model the answer?

**DOI:** 10.1108/JHOM-01-2018-0020

**Published:** 2018-08-12

**Authors:** Jonathan Erskine, Michele Castelli, David Hunter, Amritpal Hungin

**Affiliations:** 1 Durham University, Stockton-on-Tees, UK; 2 Institute of Health and Society, Newcastle University, Newcastle upon Tyne, UK

**Keywords:** Integrated clinical care, Mayo Clinic, Multi-specialty hospital care, NHS hospitals

## Abstract

**Purpose:**

The purpose of this paper is to determine whether some aspects of the distinctive Mayo Clinic care model could be translated into English National Health Service (NHS) hospital settings, to overcome the fragmented and episodic nature of non-emergency patient care.

**Design/methodology/approach:**

The authors used a rapid review to assess the literature on integrated clinical care in hospital settings and critical analysis of links between Mayo Clinic’s care model and the organisation’s performance and associated patient outcomes.

**Findings:**

The literature directly concerned with Mayo Clinic’s distinctive ethos and approach to patient care is limited in scope and largely confined to “grey” sources or to authors and institutions with links to Mayo Clinic. The authors found only two peer-reviewed articles which offer critical analysis of the contribution of the Mayo model to the performance of the organisation.

**Research limitations/implications:**

Mayo Clinic is not the only organisation to practice integrated, in-hospital clinical care; however, it is widely regarded as an exemplar.

**Practical implications:**

There are barriers to implementing a Mayo-style model in English NHS hospitals, but they are not insurmountable and could lead to much better coordination of care for some patients.

**Social implications:**

The study shows that there is an appetite among NHS patients and staff for better coordinated, multi-specialty care within NHS hospitals.

**Originality/value:**

In the English NHS integrated care generally aims to improve coordination between primary, community and secondary care, but problems remain of fragmented care for non-emergency hospital patients. Use of a Mayo-type care model, within hospital settings, could offer significant benefits to this patient group, particularly for multi-morbid patients.

## Introduction

Despite attempts to provide complete and integrated services in English National Health Service (NHS) hospitals, many patients seem to receive fragmented and episodic care, resulting in multiple hospital attendances, reviews and shuffling between specialities. This cannot be good for patients or for efficiency. We aimed to compare models of care between an organisation reported to provide high-quality integrated clinical care (Mayo Clinic) and the situation in the English NHS, and to draw lessons which could improve patient care as well as patient and staff satisfaction.

Recent reforms to the English NHS have included a strong focus on integrated care (IC) models, which are largely concerned with integration of care across primary, community and secondary care, and between the health and social care systems. While such programmes may improve coordination between the traditional silos of health and social care, encourage multidisciplinary approaches to patient care and offer at least the possibility of cost savings, they do not directly address a pernicious issue within the secondary care sector: the episodic and uncoordinated care of non-emergency patients in NHS hospitals, particularly in the case of complex, multi-morbid patients. In this paper we argue that there may be a strong case to embed (or return to) a culture and practice of bringing multi-specialty expertise around the patient, to offer more holistic and timely diagnosis and intervention, within the setting of NHS hospitals.

In 1997 the Labour Government published a white paper “The new NHS: modern, dependable” which marked a turning point in the organisation of healthcare in the English NHS (Department of Health, 1997). It proposed to abolish the “internal market” in favour of IC, and to invest the supposed savings in targeted service improvements and an expanded focus on population health. Since 1997, government policy has continued to promote better integration of care, for reasons which have been claimed to include cost saving, improved performance and quality, patient safety and satisfaction, a need to respond to changing health and social care technologies and the professional views of clinical staff and service managers (Department of Health, 2008; Ham and Curry, 2011; Gretton and Honeyman, 2016; Monitor, 2014).

The most recent major reorganisations of the English NHS, implemented by the 2010–2015 UK Coalition Government, have carried forward this ambition, with the 2010 health white paper “Equity and excellence: liberating the NHS” arguing for “putting patients in control” and for full integration across health and social care (Department of Health, 2010). In the period between the publication of this white paper and the passing of the Health and Social Care Act, a number of influential health policy organisations published reports which were broadly supportive of this aim (UK Government, 2012; The King’s Fund, 2012, 2013; Goodwin *et al.*, 2012). Integrated, patient-centred care continued to be the focus of a number of studies and reports published up to, and following, the launch of NHS England’s Five Year Forward View (5YFW) in 2014, adding to the sense that IC was the only game in town (Royal College of General Practitioners, 2014; NHS England, 2014).

The 5YFW argued that these changes to NHS structures and practices are broadly necessary to achieve the triple aim of improving the patient experience and population health, and reducing the per capita cost of healthcare. To this end, the 5YFW proposed clear commitments to breaking down barriers in how care is provided, and developing new, integrated models of care and new relationships with patients and communities. These commitments are now embodied in initiatives such as the new care models (and associated “Vanguard” sites), the sustainability and transformation partnerships (STPs) and the emerging accountable care systems (ACSs) and accountable care organisations (ACOs) (NHS England, 2017a, b; Ham *et al.*, 2015; Imison, 2015; The King’s Fund, 2017).

The new care models which are developing throughout the NHS in England have a common theme: each proposes some form of integration which spans traditional silos, between primary, community and secondary care and/or between the healthcare and social care systems. While these models undeniably aim to remove barriers to patient-centred, IC, they still assume that for non-emergency treatment of patients who need specialist care or diagnosis in a hospital environment, the English NHS will continue to work through a hub and spoke model (NHS England, 2017a, b), with general practitioners (GPs) acting as gatekeepers. Patients will continue to be offered an appointment with a hospital consultant in a particular department, which may take place only after weeks or months (NHS Choices, 2017). Following the hospital appointment, patients will generally have to arrange another visit to the GP to review the specialist’s advice, begin treatment and often then to find out what further advice, care or testing may be required. If there is a need to return to the same specialist, to be seen by a different one, or to carry out other tests, patients may need to return for a further appointment, arranged by either the GP or the hospital specialist.

Within this episodic model, patients with complex multiple conditions may continue to find themselves spending long periods waiting to see the next clinician in the chain, often unsure which physician ultimately has oversight of and responsibility for their care. Waiting for specialist advice, and uncertainty concerning the coordination of care, may be sources of anxiety and stress for patients—particularly when appointments or procedures are postponed or cancelled due to other pressures within the health system (NHS England, 2016). Furthermore, the NHS patient population contains an increasing number of such multi-morbid individuals (Department of Health, n.d.), who often need to be assessed by a range of different hospital specialists, sometimes because a first appointment reveals a previously undiagnosed, untreated or under-treated condition, because the patient simply requires expert advice and treatment from a different specialist or sub-specialist, or because of lowered thresholds for diagnosis (Starfield, 2011; Rowland and Paddison, 2013). In essence, there is a growing mismatch between the increasing complexity and acuity of many patients requiring non-emergency care in a hospital setting, and a system still largely designed to treat them in a linear, episodic manner.

For these patients, it could be very beneficial to organise care differently within the hospital environment, such that they would have rapid access to a range of specialist opinion and diagnostic procedures which could address multiple care needs in a short space of time. In 2016, a team of researchers from Durham University’s School of Medicine, Pharmacy and Health set out to find examples of hospital organisations which organise diagnosis and care in a more holistic way, using multidisciplinary teams of clinician specialists, particularly for patients with complex conditions and/or multi-morbidities. An early, rapid scan of the literature showed that one of the best-known examples of such an organisation is Mayo Clinic in the USA, which reports itself as being organised around a highly patient-centric care model, based on multi-specialty practice and which makes extensive use of a “coordinating physician” to manage the patient’s care during elective, outpatient or ambulatory episodes of care (Mayo Clinic, 2014). Other hospital organisations in the USA have also adopted some elements of hospital-based, integrated clinical care, based on the use of the “hospitalist” doctor model which emerged in the late 1990s (Wachter, 1999). However, Mayo Clinic is reported to have operated this type of care model for many decades and could therefore be regarded as an exemplar organisation.

The same scan of the literature confirmed that Mayo Clinic is frequently cited as one of the highest performing hospital organisations in the USA (Kodner and Spreeuwenberg, 2002; NHS England, 2015). For that reason, coupled with the distinctive approach to the organisation and management of patient care within the hospitals operated by Mayo Clinic, the research team considered that there would be merit in pursuing a two-stage action research project, to investigate whether there are aspects of the Mayo model which could be adopted within English NHS hospitals in order to address the continuing challenge of episodic and fragmented patient care, as outlined earlier in this section. To this end, the team proposed to carry out the following phases of work:

1. Conduct a rapid review of the literature into the Mayo Clinic’s model of care, with particular focus on evidence concerning any links between the “Mayo Model” and patient outcomes, staff and patient experience, hospital performance data and finances.
2. Carry out qualitative research within an English Foundation Trust hospital to investigate the potential barriers to implementing a care model similar to that used by Mayo Clinic.

The overall aim of this study is to determine whether there are aspects of the distinctive Mayo Clinic model of care which could be translated into hospital settings within the English NHS, in order to overcome the fragmented and episodic nature of non-emergency patient care.

This paper reports on the first phase of the study. The findings from Phase 2 of the research, including a fact-finding tour of Mayo Clinic in May 2016, are to be separately reported in a forthcoming article.

The research study protocol was granted ethical approval by the Ethics Committee of Durham University’s School of Medicine, Pharmacy and Health in April 2015.

## Method

We employed a rapid review method to examine the literature on the Mayo Clinic model and other models of hospital-based ICC. Rapid reviews have become a common means of obtaining rigorous but relatively quick insights into the state of knowledge and evidence relating to a particular field of enquiry (Khangura *et al.*, 2012). They are popular with policy makers and public-sector institutions, as they can be more responsive to the timeframes encountered when translating from policy development to implementation (National Collaborating Centre for Methods and Tools, 2012). This streamlined approach to synthesising evidence has been particularly welcomed by decision makers in healthcare settings, owing to the rapidly evolving landscape of health and healthcare systems (Khangura *et al.*, 2012). Systematic reviews may be the “gold standard” under some circumstances, but they generally require significant resources over a 6- to 24-month timeframe, and they often focus on a tightly defined clinical question. Neither of these conditions applied to our study.

Our rapid review was carried out in late 2016. The principal aim was to locate peer-reviewed articles, books, “grey” literature, reports and any other material concerning the implementation and use of hospital-based, ICC models in general, and the Mayo Clinic’s model of care in particular. In addition to the rapid review, we also consulted some literature reviews and syntheses concerning the general concept of IC in the English NHS and other health systems, to discover whether these included discussions of hospital-based ICC, but also to establish a context for the more generic use of IC in health system research.

For the rapid review, we used the following search terms:

- “integrated clinical care” (in title only);
- “Hospital”+(“integrated clinical care” OR “hospitalist”) (in abstracts);
- “NHS”+(“integrated clinical care” OR “hospitalist”) (in abstracts); and

- (“Mayo Clinic” OR “Mayo Model”)+(“integrated care” OR “integrated clinical care”; OR “coordinated care” OR “multidisciplinary care” OR “hospitalist”) (in abstracts).

The search terms were chosen to reflect our particular interest in the Mayo Clinic model of care and ICC in a hospital setting, but with some additional terms included to reflect some of the most commonly used descriptors which are close in meaning to “integrated clinical care”. The term “hospitalist” was included because it is commonly used in the USA to describe a class of in-hospital physician who is charged with medical management of patients while they are present within the hospital environment (Wachter, 1999).

The rapid review was carried out by two members of the research team, and the search terms were used to interrogate the following sources:

- Academic databases: Medline, Web of Science and Google Scholar (the latter for background material on IC in the English NHS).
- Websites of authoritative, UK-based organisations known to have conducted research into IC, including the King’s Fund, the Health Foundation and the Nuffield Trust.
- “Grey” literature sources.

The literature search was updated in early 2017 to capture any peer reviewed or other relevant literature that was published during Phase 2 of the project. The researchers also consulted other members of the research team, who have in-depth, expert knowledge of the field, to sense check the results of the searches and to ensure that there were no omissions.

## Results

Table I summarises the results of the literature search in relation to peer-reviewed journal articles potentially relevant to hospital-based ICC. Table II presents information on publications specifically concerned with the Mayo model of care.

Table I shows that a search on “integrated clinical care”, whether associated with Mayo Clinic or not, produces very few results of any relevance.

There were 95 results from Medline and 108 from Web of Science, when searching on (integrated + clinical + care). However, on reading the abstracts associated with these results, the articles were found to focus on integrated clinical pathways across primary, secondary, community and healthcare, rather than on multi-specialty, IC solely within a hospital setting.

There were only six results from (Mayo + Clinic) + (integrated + care), and these were concerned with change strategies, the application of team-based care to ACOs, creating a quality framework in a clinical environment, managing relations between physicians and hospital organisations in the USA and use of centralised patient records in a team-based clinical environment. The use of (Mayo + model) as search terms yielded only three further results, which concerned a single specialty within Mayo Clinic, care coordination across inpatient and outpatient settings and the patient experience of community-based healthcare.

There was also scant evidence concerning the use of hospitalists in the Mayo Clinic, with just one article concerning the work of hospitalists with teams of orthopaedic surgeons and another which cites the work of hospitalists with Mayo Clinic geriatricians.

When the search terms were widened to include Hospital + (integrated + clinical + care), although a large number of results were returned, these could be broadly grouped into articles concerning the integration of professional groups into hospital organisations, integration between hospital, primary and community care organisations, “product lines” within hospitals, overcoming professional silos, integration of financial and IT systems, hub/spoke hospital models, integration of specific specialties (e.g. ICU and palliative care) or managed care programmes in and out of hospital.

The rapid review of literature which directly addresses the Mayo Clinic model of care (Table II) reveal only two peer-reviewed journal articles (Viggiano *et al.*, 2007; Berry and Beckham, 2014), and these are largely descriptive accounts. Other literature comprises two books (Mayo Clinic, 2014; Berry and Seltman, 2008) and a small number of reports produced by professional associations or healthcare policy and practice foundations such as the Nuffield Trust, the Royal College of General Practitioners, the Commonwealth Fund and the Mayo Foundation (Casalino, 2011; Royal College of General Practitioners, 2014; Curtright *et al.*, 2000).

## Discussion

This section covers two areas: the background and context of IC as recently understood in the English NHS, and the Mayo Clinic model of hospital-based ICC.

### Integrated care: background and context in the English NHS

Our rapid literature review on the context of IC in the English NHS found that the most recent conceptualising of “IC” focuses on the creation of healthcare delivery systems which span some of the traditional silos of care (Casalino, 2011; Shaw *et al.*, 2011; Kodner and Spreeuwenberg, 2002). That is, IC is taken to refer to processes of coordinating patient care between hospital, primary, community and social care, often with the aim of preventing unnecessary recourse to specialist services, and with aspirations to improve system efficiency (including hospital bed utilisation), productivity and the quality of care. In this vein, in the 2008 report “High quality care for all” Darzi suggests:

[…] more integrated services for patients, by piloting new integrated care organisations, bringing together health and social care professionals from a range of organisations – community services, hospitals, local authorities and others, depending on local needs

(Department of Health, 2008, p. 13).

In 2010, the Nuffield Trust and the King’s Fund jointly published a comprehensive synthesis of knowledge concerning the typologies of integration, the nature of different types of IC organisations and how they could operate in the context of English health and social care systems, considerations of how IC might bridge the commissioner-provider separation, and the major challenges to integrated care organisations (ICOs) becoming mainstream institutions (Lewis *et al.*, 2010). Three types of ICO are envisaged: networks of provider organisations; merged organisations which could join up different care sectors; and integrated commissioner-provider organisations. The typologies of IC are categorised as organisational (mergers, collectives and coordinated networks), functional (integration of non-clinical support and administrative functions), service-based (multidisciplinary teams within individual clinical services), clinical (care provided as a coherent process across professional groupings), normative (a shared ethos and values) and systemic (coherence of policies and approach at all organisational levels).

All of the above reports, in addition to others which address specific pilot projects in the English NHS (Nuffield Trust, 2013), focus on types of integration which are concerned with improving coordination between health and social care, or between different healthcare provider organisations. None of them addresses directly the kind of hospital-based ICC that is the focus of our research project.

### Mayo Clinic model of care

The Mayo Clinic model of care is described in an eponymous document published by the Mayo Foundation for Medical Education and Research (Mayo Clinic, 2014). Approved by the Mayo Clinic Boards of Governors, it is written as a manifesto and as a guide to the culture of the organisation, covering various aspects of expected behaviours of staff, commitments to high-quality, professional practice and ambitions for innovation and clinical excellence. In relation to patient care, the document focuses on the following:

- collegial, cooperative, staff teamwork with true multi-speciality integration;
- an unhurried examination with time to listen to the patient;
- physicians taking personal responsibility for directing patient care over time in a partnership with the local physician;
- highest quality patient care provided with compassion and trust;
- respect for the patient, family and the patient’s local physician;
- comprehensive evaluation with timely, efficient assessment and treatment; and
- availability of the most advanced, innovative diagnostic and therapeutic technology and techniques.

Given this long-standing mission statement, and Mayo Clinic’s preeminent reputation among US hospital organisations, it is perhaps surprising that our literature review, as summarised in Tables I and II, found only two peer-reviewed articles which directly assess the relationship between Mayo Clinic’s highly integrated, patient-centred model of care and overall efficacy, productivity or cost-effectiveness of the organisation, patient outcomes or patient and staff satisfaction. It is the case that there is a very extensive literature, much of it originating from *Mayo Clinic Proceedings* (a peer-reviewed clinical journal, sponsored by Mayo Clinic), concerning Mayo Clinic’s medical and surgical research in a very broad range of specialities, but this focuses on clinical trials data and findings related to specific interventions; it does not relate in any direct sense to Mayo Clinic’s distinctive model of care—how it is organised, operates and its impact.

From Table II we can see that information, analysis and opinion concerning the “Mayo Model” are largely to be found in a small number of books and reports, as well as a number of articles in the “grey” literature, often within the area of management studies. A key example of such a source is the widely cited book *Management Lessons from Mayo Clinic*, written to showcase Mayo Clinic’s distinctive organisational ethos and culture in order to provide instructive guidance for managers operating outside the healthcare sector (Berry and Seltman, 2008). The authors, one of whom occupies a senior marketing position at Mayo Clinic, provide a history of the organisation from its inception up to 2008, emphasising the development of a healthcare organisation oriented towards patient (customer) satisfaction and high-quality care, and which makes extensive use of technologies and processes to optimise information flow and communication between clinicians, managers, administrators and the patient. It examines Mayo Clinic’s systems for developing leadership potential, and the determined yet conservative growth of Mayo Clinic as a brand. The authors acknowledge the consistently high performance of Mayo Clinic’s specialties, as judged by various indexes based in the USA, and claim that this achievement is directly linked to the organisation’s model of care, and to its non-profit commitment to investing heavily in research and staff development (US News Health, 2017). According to many measures, the Mayo Clinic is a high-performing healthcare organisation, but the authors do not offer a critical analysis or comparison of Mayo Clinic with other US-based healthcare providers, some of which are clearly comparable according to a number of organisations which rank US hospital established hospital performance (US News Health, 2017). It is therefore difficult to conclude definitively, from the evidence presented, that Mayo Clinic’s demonstrably excellent patient care is necessarily achieved through the adoption of its particular care model.

A 2009 Commonwealth Fund report (McCarthy *et al.*, 2009), one of 15 case examples used to illustrate six attributes of an ideal healthcare delivery system, is a comprehensive account of both Mayo Clinic and Mayo Health System (the regional affiliate of Mayo Clinic). The report rates Mayo Clinic highly against the six attributes of information continuity, care coordination and transitions, peer review and teamwork for high-value care, continuous innovation and easy access to appropriate care. The analysis of Mayo Clinic’s success in implementing a highly integrated and patient-centred care model emphasises the clinician-led system of governance, peer accountability, the importance of an advanced electronic medical record system and infrastructure which is highly supportive of clinical work.

This report focuses on a number of the distinctive features of Mayo Clinic, including employment of salaried physicians, continuous investment in technical and organisational infrastructure and governance arrangements which are led by physicians who have been trained to make decisions based on patient need. However, the key theme running through the report is that of collaborative, highly integrated, multidisciplinary teamwork. This theme appears in discussions of care coordination and transitions, systems for peer review, continuous service improvement, procurement of new technologies and performance indicators. The authors describe the Mayo Clinic’s approach to team-based care coordination, thus:

[…] physicians from every medical speciality work collaboratively to meet individual patient needs, often during the same patient visit […]. Every Mayo patient is assigned a coordinating physician whose job is to ensure that the patient has an appropriate plan of care, that all ancillary services and consultations are scheduled in timely fashion to meet the patient’s needs, and that the patient receives clear communication throughout […]

(McCarthy *et al.*, 2009, p. 5).

While the Commonwealth Fund report provides a clear account of Mayo Clinic’s achievements in a range of clinical and non-clinical areas, much of the source material is derived from Mayo Clinic itself, either through contacts with staff or by reference to articles contained in the in-house journal *Mayo Clinic Proceedings* or hosted by the Mayo Clinic website. There are a limited number of references to peer-reviewed academic journals, and where these are cited they relate to articles which focus on studies concerning very specific, narrowly defined interventions—for example, the impact of “open access scheduling”, the treatment of asthma patients or community-based diabetes care.

Our literature review found only two peer-reviewed articles published in journals other than *Mayo Clinic Proceedings* which directly addresses the Mayo Model and its potential contribution to the success of Mayo Clinic in its three locations across the USA. In particular, we did not find any comparative studies which, for example, might assess the contribution of Mayo Clinic’s professional and organisational culture in areas such as staff development and retention, patient satisfaction or patient outcomes, with other high-performing US-based healthcare organisations. Given the high profile and status of Mayo Clinic in the USA and internationally, as well as current high levels of interest in IC in general, it is perhaps surprising that this area has not received more attention.

Where authors do attempt a critical examination of the Mayo Model, they are often highly reliant on articles which are published by *Mayo Clinic Proceedings,* or which are found in the business management literature. For example, in “Putting the needs of the patient first: Mayo Clinic’s core value, institutional culture, and professionalism covenant” (Viggiano *et al.*, 2007), out a list of 20 citations, ten publications fall into these two categories. Berry and Beckham present an argument for Mayo Clinic’s ethos of team-based medicine as providing at least part of the solution to the conundrum faced by many health economies in England: trying to overcome the existing professional silos which stand in the way of IC in the service of a whole patient population. However, in doing so, the authors rely mostly on literature concerning the development of ACOs; references to the Mayo’s organisational structures, such as highly integrated teamwork and sophisticated communications systems, are restricted to one book and an online article (Berry and Beckham, 2014).

We also sought to unearth literature which more generally discusses what we have termed “hospital-based ICC”, similar to that practised by Mayo Clinic, in the hope of finding examples in operation elsewhere in the world. In brief, we found many cases of hospital organisations which make use of: multidisciplinary teams in some or all of their care pathways; vertical integration with primary and/or community health professionals; horizontal integration with partner organisations, to improve service resilience and choice; inclusion in population-based health systems which span health and social care and prevention of ill health. Many of these examples reflect the proposed models of care which are outlined in the NHS 5YFW and in subsequent guidance on its implementation (NHS England, 2015). Others can be found in recent literature on organisations such as Jönköping in Sweden or the integrated health and social programme which is currently underway in the South Karelia region of Finland (Baker *et al.*, 2008; Karhula *et al.*, 2014).

## Conclusions

Within the context of the English NHS, “IC” is a loosely defined term which has become associated with a number of different approaches to bringing about organisational, clinical, cultural and financial reconfiguration and reform, mainly in relation to better communication between, and coordination of, staff working within healthcare and/or between health and social care. For the purposes of our research study, however, we are interested in integrated clinical care within a hospital setting which allows physicians from a range of specialties to focus their attention, in as efficient a way as possible, on the holistic health needs of their patients, supported by non-clinical colleagues and effective technologies.

The forms of integration currently being developed in the English NHS through the new care models (Vanguards), STPs, ACSs and ACOs may (or may not) lead to better patient outcomes, improved patient and staff satisfaction and greater productivity, but they do not appear to address directly our focus of interest: the patient’s journey specifically within the hospital setting, following referral to a hospital specialist. In particular, it is not clear at present if any of the proposed models of IC will overcome the episodic and fragmented pathways within hospitals, experienced by patients with chronic illness or multi-morbidities—frequently elderly people and the most vulnerable patients.

Mayo Clinic has reportedly developed an organisational culture and associated processes which ensure that most patients are attended by a multi-specialty group of clinicians who collectively address the “whole patient”, managed by a coordinating physician. A small number of authors and commentators have identified a range of key enabling factors in this model, as summarised in Table III. These authors and commentators have not provided evidence of original independent empirical academic research into the efficacy of the Mayo model and, on the basis of our rapid literature review, it would be difficult to reach definitive conclusions about which elements of Mayo Clinic’s practice apparently have led to success in terms of the quality of care provided to patients. Nonetheless, the anecdotal evidence overwhelmingly suggests that Mayo Clinic’s clinical model could address our research focus: the patient journey on referral to a hospital specialist and the management of multi-morbid, chronically ill patients within the hospital environment.

Furthermore, it is arguably the case that, in the context of English NHS hospitals, some of these factors are clearly present in most English NHS hospitals, while others are less well represented or currently absent, as indicated in Table III.

If the Mayo Model offers non-emergency, multi-morbid patients a more holistic, multi-specialist and highly coordinated approach to their care, achieving high-quality outcomes and greater patient and staff satisfaction, Table III suggests that a trial of the Mayo Model in the English NHS would require development of two key staff competencies:

1. Specialist hospital doctors with the means and authority to collaborate with colleagues in other specialities to provide advice and guidance in a timely way, in order to provide a highly patient-centred care plan which takes account of the totality of the patient’s needs.
2. Administrators who are solely tasked with obtaining optimal, in-hospital patient flow, including the scheduling of patient appointments, testing and diagnostic procedures and timely, error-free provision of clinical information.

In addition, a Mayo Model for the English NHS would also require significant further investment in information and communication technology, to ensure near real-time access to diagnostic information and to enable the closest possible coordination with primary and community care clinicians, before and following hospital visits.

We therefore argue that it would be worthwhile, assuming that the results of Phase 2 of our research study do not suggest insurmountable barriers, to test this model in an NHS hospital environment, and to investigate whether it could lead to improved outcomes for patients, greater resource efficiencies and, potentially, cost savings overall. The key enabling factors of the Mayo model, as described above, could provide a testable framework against which to evaluate the success, or otherwise, of such a pilot project.

## Figures and Tables

**Table I:** Results from Medline and Web of Science databases concerning peer-reviewed journal articles relevant to the Mayo model and hospital-based ICC

Search terms	No. of results	No. of result relevant to hospital-based ICC
*Medline*		
Integrated + Clinical + Care (title only)	95	0
(Mayo + Clinic) + (Integrated + Care)	32	6
(Mayo + Clinic) + (Integrated + Clinical + Care)	3	0
(Mayo + Clinic) + (Coordinated + Care)	7	0
(Mayo + Clinic) + (Multidisciplinary + Care)	16	0
(Mayo + Clinic) + Hospitalist	2	2
(Mayo + Model) + (Integrated + Care)	37	5
(Mayo + Model) + (Integrated + Clinical + Care)	3	0
(Mayo + Model) + (Coordinated + Care)	4	0
(Mayo + Model) + (Multidisciplinary + Care)	16	0
(Mayo + Model) + Hospitalist	0	0
Hospital + (Integrated + Clinical + Care)	1,127	n/a
Hospital + Hospitalist	505	n/a
NHS + (Integrated + Clinical + Care)	82	0
NHS + Hospitalist	2	0
*Web of Science*		
Integrated + Clinical + Care (title only)	108	0
(Mayo + Clinic) + (Integrated + Care)	29	n/a
(Mayo + Clinic) + (Integrated + Clinical + Care)	12	0
(Mayo + Clinic) + (Coordinated + Care)	10	0
(Mayo + Clinic) + (Multidisciplinary + Care)	19	0
Mayo + Clinic + Hospitalist	1	1
(Mayo + Model) + (Integrated + Care)	18	n/a
(Mayo + Model) + (Integrated + Clinical + Care)	6	0
(Mayo + Model) + (Coordinated + Care)	6	0
(Mayo + Model) + (Multidisciplinary + Care)	4	0
Mayo + Model + Hospitalist	2	0
Hospital + (Integrated + Clinical + Care)	2,410	n/a
NHS + (Integrated + Clinical + Care)	124	0
NHS + Hospitalist	2	0

**Table II:** Literature describing the Mayo Clinic model of care (from all literature search sources)

Author	Title	Publisher	Year
Mayo Clinic	Mayo Clinic model of care	Mayo Clinic	2014
Berry *et al.*	Care coordination for patients with complex health profiles in inpatient and outpatient settings	*Mayo Clinic Proceedings*	2013
McCarthy *et al.*	Mayo Clinic: multidisciplinary teamwork, physician-led governance, and patient-centred culture drive world-class health care	Commonwealth Fund	2009
Viggiano *et al.*	Putting the needs of the patient first: Mayo Clinic’s core value, institutional culture, and professionalism covenant	*Academic Medicine*	2007
Berry and Beckham	Team-based care at mayo clinic: a model for ACOS	*Journal of Healthcare Management*	2014
Royal College of General Practitioners	An Inquiry into patient-centred care in the 21st Century	Royal College of General Practitioners	2014
Curtright *et al.*	Strategic performance management: development of a performance measurement system at the Mayo Clinic	*Journal of Healthcare Management*	2000
Casalino	GP commissioning in the NHS in England: ten suggestions from the United States	Nuffield Trust	2011
Berry and Seltman	*Management lessons from the Mayo*	McGraw-Hill	2008

**Table III:** Key enabling factors of the Mayo model of care

Mayo Clinic	English NHS hospitals
Operation as a non-profit healthcare organisation	Present
Employment of salaried physicians	Present
Strong commitment to clinical research, training and clinical leadership development	Present
Use of a coordinating physician with responsibilities to ensure that the patient is attended, in a timely manner, by every clinical and non-clinical professional who may contribute to the patient’s care	Not present
Investment in the most up-to-date electronic health record systems and rapid, high quality digital communications	Varies across the English NHS; in development
Use of staff whose sole purpose is to schedule appointments, testing and diagnostic procedures, and to ensure timely, error-free information flow	Not present
Well-developed partnerships between Mayo Clinic staff and patients’ family physicians, with extensive sharing of information and data	Varies across the English NHS; in development
Mutual respect between patients and hospital staff	Present
